# The Role of Pyoluteorin from *Pseudomonas protegens* Pf-5 in Suppressing the Growth and Pathogenicity of *Pantoea ananatis* on Maize

**DOI:** 10.3390/ijms23126431

**Published:** 2022-06-09

**Authors:** Qin Gu, Junqing Qiao, Ruoyi Wang, Juan Lu, Zhengqi Wang, Pingping Li, Lulu Zhang, Qurban Ali, Abdur Rashid Khan, Xuewen Gao, Huijun Wu

**Affiliations:** 1 Key Laboratory of Integrated Management of Crop Diseases and Pests, Ministry of Education, Department of Plant Pathology, College of Plant Protection, Nanjing Agricultural University, Nanjing 210095, China; guqin@njau.edu.cn (Q.G.); 2019102046@njau.edu.cn (R.W.); lujuan726@163.com (J.L.); wangzhengqi233@163.com (Z.W.); liping18351000355@163.com (P.L.); 18795971373@163.com (L.Z.); 2020202068@stu.njau.edu.cn (Q.A.); malix.477@gmail.com (A.R.K.); gaoxw@njau.edu.cn (X.G.); 2 Institute of Plant Protection, Jiangsu Academy of Agricultural Sciences, Nanjing 210014, China; qiaojunqing@jaas.ac.cn

**Keywords:** *Pseudomonas protegens*, *Pantoea ananatis*, pyoluteorin, orfamide, biofilm, biocontrol

## Abstract

The rhizospheric bacterium *Pseudomonas protegens* Pf-5 can colonize the seed and root surfaces of plants, and can protect them from pathogen infection. Secondary metabolites, including lipopeptides and polyketides produced by Pf-5, are involved in its biocontrol activity. We isolated a crude extract from Pf-5. It exhibited significant surface activity and strong antibacterial activity against *Pantoea ananatis* DZ-12, which causes maize brown rot on leaves. HPLC analysis combined with activity tests showed that the polyketide pyoluteorin in the crude extract participated in the suppression of DZ-12 growth, and that the lipopeptide orfamide A was the major biosurfactant in the crude extract. Further studies indicated that the pyoluteorin in the crude extract significantly suppressed the biofilm formation of DZ-12, and it induced the accumulation of reactive oxygen species in DZ-12 cells. Scanning electron microscopy and transmission electron microscopy observation revealed that the crude extract severely damaged the pathogen cells and caused cytoplasmic extravasations and hollowing of the cells. The pathogenicity of DZ-12 on maize leaves was significantly reduced by the crude extract from Pf-5 in a dose-dependent manner. The polyketide pyoluteorin had strong antibacterial activity against DZ-12, and it has the potential for development as an antimicrobial agent.

## 1. Introduction

The phytopathogenic *Pantoea ananatis* is a Gram-negative bacterial species that causes severe economic losses in onion, maize, pineapple, and honeydew melon [1,2]. *P. ananatis* can also cause brown rot in maize leaves and cause economic losses in maize production [2]. The disease, termed maize brown rot leaves, produces water-soaked lesions on the leaves, which eventually become necrotic and straw-colored. The disease can be disseminated over the entire leaf surface in its advanced stage [3]. This disease also causes premature leaf drying, shortening of the growth cycle, and the reduction of kernel length and weight [4].

The plant rhizosphere is the narrow zone of soil that is relatively rich in nutrients due to the accumulation of a variety of plant exudates [5]. The rhizosphere harbors beneficial bacteria named plant growth-promoting rhizobacteria (PGPR). These colonize the rhizosphere, root surfaces, and radicular tissues. They promote plant growth and suppress soil-borne plant pathogens [6]. These beneficial biological activities have led to the development of many PGPR strains as commercial biological control agents (BCAs). Among these PGPR strains, *Pseudomonas* species have received significant attention [7].

To survive and to successfully compete with other microorganisms, PGPR can effectively utilize seed and root exudates to produce a wide spectrum of bioactive metabolites such as antibiotics, siderophores, volatile organic compounds (VOCs), and growth-promoting substances [8]. The *Pseudomonas* biocontrol strains also can induce systemic resistance in plants [9]. Several *Pseudomonas* strains, such as *Pseudomonas protegens* Pf-5, are effective at controlling plant diseases caused by *Pythium ultimum* (damping-off of cotton) and *Rhizoctonia solani* [10]. The *P. protegens* CHA0 strain suppresses root rots of tobacco and tomato caused by *Pythium* sp., as well as the damping-off of cucumber and take-all of wheat [11]. In addition, *Pseudomonas fluorescens* 2P24 can protect plants against tomato bacterial wilt caused by *Ralstonia solanacearum* [12].

Among the *Pseudomonas* biocontrol strains, the ability to produce antibiotics is an effective method for controlling plant diseases. Phenazine-1-carboxylic acid (PCA) produced by *Pseudomonas* species provides the effective suppression of pathogens [13], such as *Gaeumannomyces graminis* which causes take-all disease of wheat [14]. PCA is the main active component of the benzimidazole-derived biopesticide “Shenqinmycin” [15]. The polyketide antibiotic 2,4-diacetylphloroglucinol (2,4-DAPG) is a phloroglucinol derivative compound from some *Pseudomonas* species that exhibits broad-spectrum antimicrobial activity [16] and suppresses black root rot of tobacco [8]. Other metabolites, such as pyoluteorin (Plt), pyrrolnitrin (Prn), siderophores, and lipopeptides are active substrates that are produced by *Pseudomonas* biocontrol agents and are reported as being associated with biocontrol activity [17,18].

The *Pseudomonas* commensal, *P. protegens* Pf-5, is notable as a BCA, for its efficient rhizosphere colonization and the ability to produce many secondary metabolites [19]. Pf-5 can produce the lipopeptides orfamide A, rhizoxin, pyrrolnitrin, pyoluteorin, 2,4-DAPG, and the siderophores pyochelin and pyoverdine [18,19,20]. These secondary metabolites contribute to the suppression of plant diseases. Pf-5 can also produce the VOC hydrogen cyanide (HCN), which is toxic to phytopathogens. In the present study, we explored the mechanism underlying the antibacterial activity of a crude extract prepared from Pf-5 against *P. ananatis* DZ-12. 

## 2. Results

### 2.1. Antibacterial and Surface Activity of Crude Extract Prepared from P. protegens Pf-5

**Figure 1:**
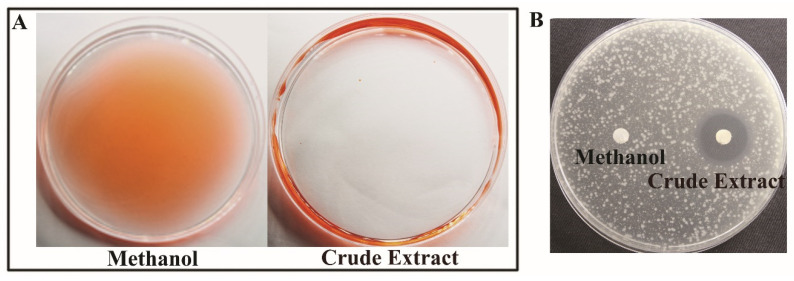
Analysis of the bio-surfactant activity prepared from *P. protegens* Pf-5. (**A**) Antibacterial activity; (**B**) Crude extract from *P. protegens* Pf-5. The crude extract was directly used.

The bio-surfactant activity of crude extract prepared from the supernatant of *P. protegens* Pf-5 was measured and expelling oil was performed according to the previously reported method [21]. The crude extract showed significant surfactant activity (Figure 1A), indicating that the surfactant compound was present in the crude extract. The crude extract also exhibited antibacterial activity against *P. ananatis* DZ-12 (Figure 1B).

### 2.2. Pyoluteorin and Orfamide A produced by P. protegens Pf-5 Showed In Vitro Antibacterial and Surface Activity, Respectively

**Figure 2:**
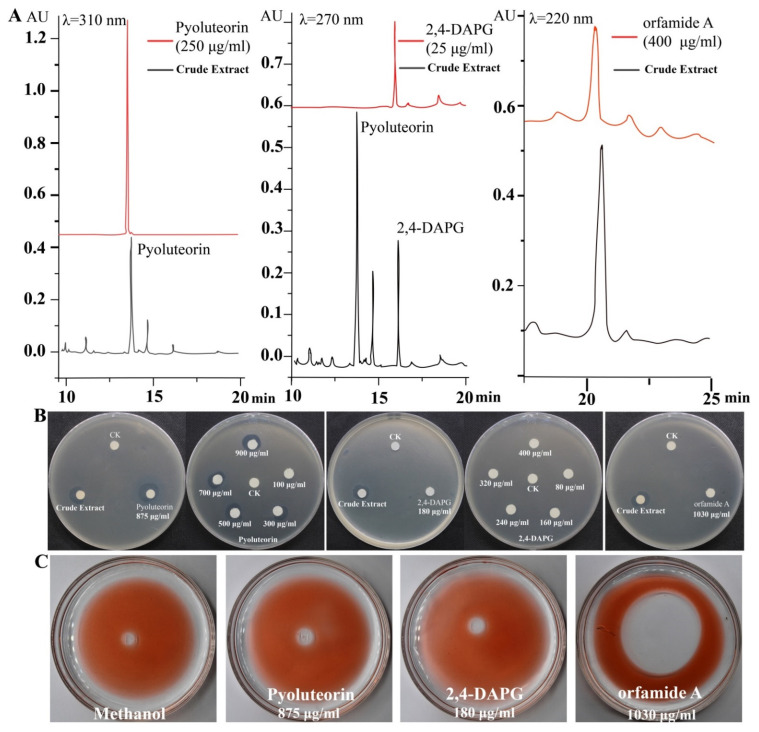
HPLC analysis of the major components in the crude extract (**A**) and detection of their antibacterial activity (**B**) and biosurfactant activity (**C**).

We identified the active compounds in the crude extract using HPLC analysis. Three different standard compounds, pyoluteorin, 2,4-DAPG, and orfamide A, were used as references. We found that these three compounds were present in the crude extract (Figure 2A). Standard curves were used to determine that the concentrations of pyoluteorin, 2,4-DAPG, and orfamide A in crude extract were 875 μg/mL, 180 μg/mL, and 1030 μg/mL, respectively. Pyoluteorin showed strong antibacterial activity among the selected compounds being treated with the same concentration (875 μg/mL) against *P. ananatis* DZ-12. However, 180 μg/mL 2,4-DAPG and 1030 μg/mL orfamide A had no significant antibacterial activity (Figure 2B). Based on these results, various doses of pyoluteorin and 2,4-DAPG were selected for further experiments. Pyoluteorin had antibacterial activity that was enhanced with increasing dose (100 to 900 μg/mL). The 2,4-DAPG had lower antibacterial activity; even at 400 μg/mL, inhibition zones were not observed (Figure 2B). To verify these results, seven mutants of Pf-5 were tested. The results showed that only mutant Δ*pltB* that cannot synthesize pyoluteorin loses its ability to suppress the growth of DZ-12 (Figure S1). These results showed that pyoluteorin was the main antibacterial compound in the crude extract. In previous findings, the crude extract also showed biosurfactant activity. We performed the same experiments and found that these three major compounds showed such activity. Orfamide A can produce expelling oil with surfactant activity. However, 2,4-DAPG and pyoluteorin did not have this activity (Figure 2C). These results demonstrate that orfamide A is the major biosurfactant in the crude extract.

### 2.3. Pyoluteorin Produced by P. protegens Pf-5 Contributes to the Inhibitory Effect on Biofilm Formation of P. ananatis DZ-12

**Figure 3:**
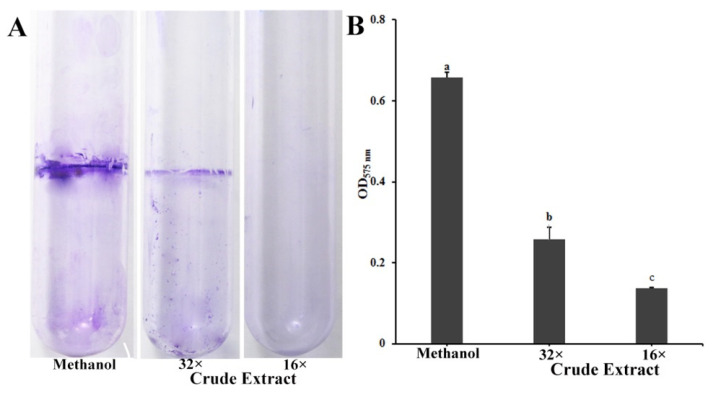
Effect of crude extract prepared from *P. protegens* Pf-5 on the biofilm formation of *P. ananatis* DZ-12. (**A**) Biofilm on the inner surface of glass tubes were stained with crystal violet and photographed; (**B**) The graph data show the average OD_575_ nm value ± standard deviation (SD) of three replicates. A concentration of 32× and 16× dilutions of the crude extract from *P. protegens* Pf-5 were used. Different letters represent statistically significant differences according to the one-way ANOVA test (*p* < 0.05).

Biofilms have a major role in plants colonization and are virulence determinants in many bacterial pathogens [22]. Because crude extract inhibits the biofilm formation of *P. ananatis* DZ-12, the biofilm was measured using crystal violet staining. Dilutions of 16× and 32× of the crude extract had a significant inhibitory effect on the biofilm formation of *P. ananatis* DZ-12 (Figure 3).

**Figure 4:**
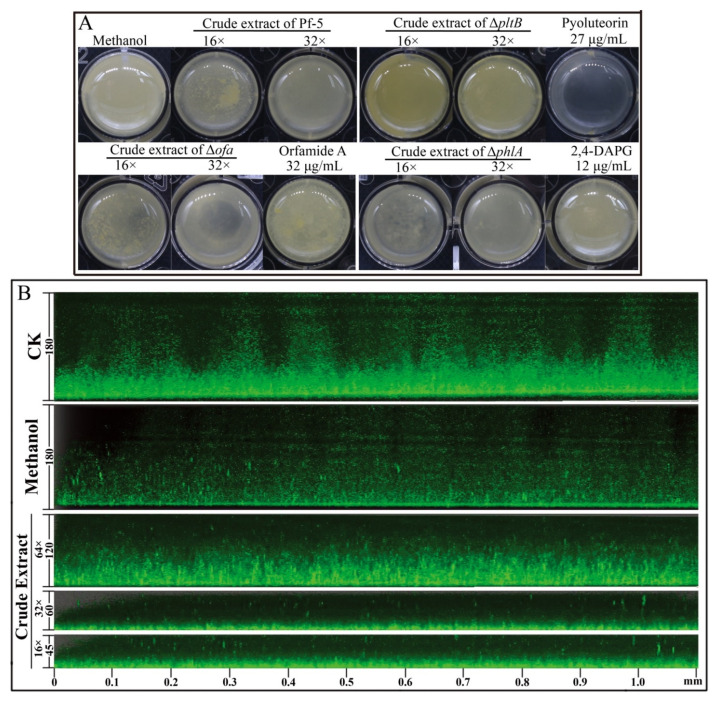
Effect of crude extract prepared from *P. protegens* Pf-5 on the biofilm formation of *P. ananatis* DZ-12. Bacterial cells were statistically cultured on LB medium in a 24-well plate (**A**), and in chambers in slides (**B**), at 28 °C for 48 h. For the common observation (**A**), different dilutions of the crude extract (32×, 16×) from *P. protegens* Pf-5 and its mutants Δ*ofa*, Δ*pltB*, and Δ*phlA* were used. A concentration of 27 μg/mL pyoluteorin, 32 μg/mL orfamide A, and 12 μg/mL 2,4-DAPG were also used. For the fluorescence microscope observation (**B**), different dilutions of the crude extract (64×, 32×, and 16×) from *P. protegens* Pf-5 were used. Representative images of the Z-stack assembly in the biofilms of cells and biofilm thicknesses were measured using a Leica SP5 confocal microscope.

In order to determine which compounds play a key role in the suppression of the biofilm of DZ-12, three mutants, Δ*ofa*, Δ*pltB*, and Δ*phlA* which have respectively lost the ability to synthesize orfamide A, pyoluteorin, and 2,4-DAPG, were used. Observations of *P. ananatis* DZ-12 biofilm in a different dilution of the crude extract from wild type Pf-5 and the two mutants Δ*ofa* and Δ*phlA* all showed suppression of biofilm formation. Their crude extract reduced the growth of bacterial cells and also disrupted the thickness of biofilm structure compared to the control methanol treatment (Figure 4A). However, mutant Δ*pltB*, which cannot synthesize pyoluteorin, has lost this activity. To confirm this phenomenon, three pure commercial standards orfamide A, pyoluteorin, and 2,4-DAPG were used. The results had showed only pyoluteorin could suppress the biofilm formation of DZ-12. This indicated that the pyoluteorin present in the crude extract was the key factor for antibiofilm activity. 

We also used *P. ananatis* DZ-12 labeled with the *gfp* gene to observe biofilm formation in detail. A similar inhibitory effect was seen under a fluorescence microscope. The thickness of biofilm was significantly reduced more than three times when *P. ananatis* DZ-12 was treated with a 16× dilution of crude extract (Figure 4B). These results further strengthen the evidence that the crude extract can inhibit the growth of *P. ananatis* DZ-12 and suppress biofilm formation.

### 2.4. Induction of Reactive Oxygen Species in P. ananatis DZ-12 Exposed to Crude Extract Containing Pyoluteorin

**Figure 5:**
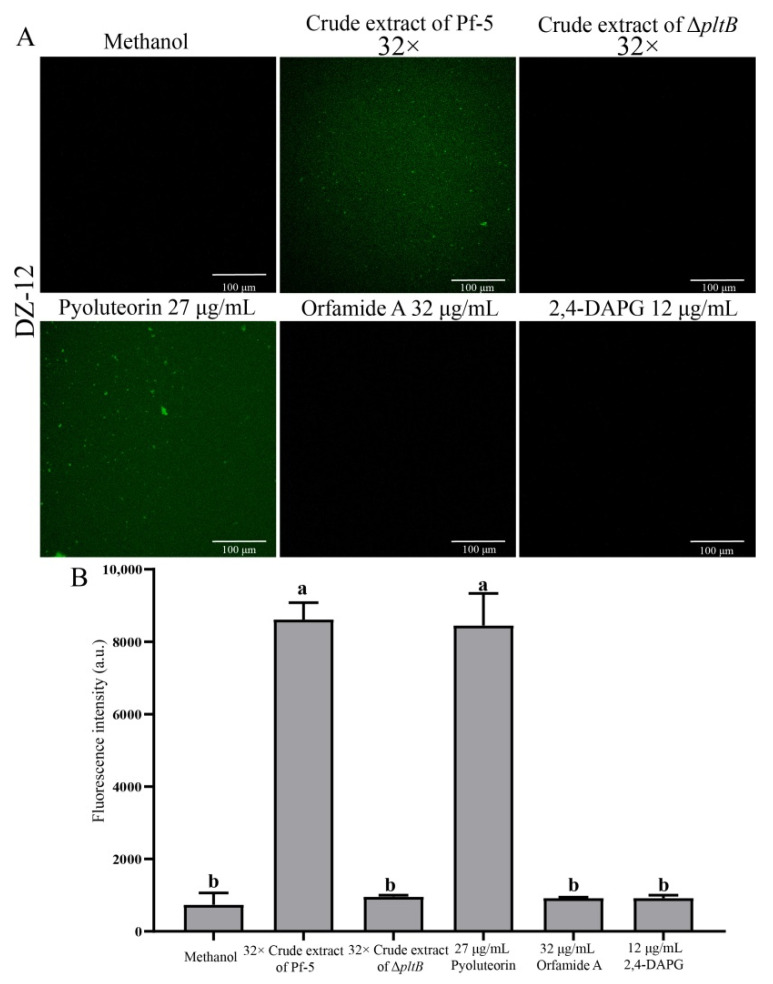
The effects of crude extract on ROS production *P. ananatis* DZ-12 observed using a Leica SP5 confocal microscope (**A**), and analyzed by EnSight Multimode Plate Reader (**B**). A concentration of 32× dilutions of the crude extract from *P. protegens* Pf-5 and its mutant Δ*pltB* were used. A concentration of 27 μg/mL pyoluteorin, 32 μg/mL orfamide A, and 12 μg/mL 2,4-DAPG were also used. The graph data show the average fluorescence intensity value ± standard deviation of three replicates. Different letters represent statistically significant differences according to the one-way ANOVA test (*p* < 0.05).

High concentrations of reactive oxygen species (ROS) production are harmful to cells and can result in cell death [23]. Our results showed that crude extract had strong antibacterial activity and induced ROS in *P. ananatis* DZ-12. Green fluorescence microscopy observation revealed that the bacterial cells had strong green fluorescence after treatment with 32 × dilutions of crude extract from Pf-5 compared to the control (Figure 5A). A similar result was also observed when cells of DZ-12 were treated with 27 μg/mL pyoluteorin (Figure 5A). However, a crude extract from the Δ*pltB* mutant, as well as orfamide A and 2,4-DAPG, did not result in the ROS accumulation of DZ-12 (Figure 5A). The fluorescence intensity confirmed that the ROS accumulation level of DZ-12, when treated with 32× dilutions of crude extract from Pf-5 and 27 μg/mL pyoluteorin, was significantly higher than that of the control, Δ*pltB*, and two compounds, orfamide A and 2,4-DAPG (Figure 5B). These results indicated that the pyoluteorin presenting in the crude extract contributes to the ROS generation of DZ-12. 

### 2.5. Ultrastructural Changes in P. ananatis DZ-12 Caused by Crude Extract

**Figure 6:**
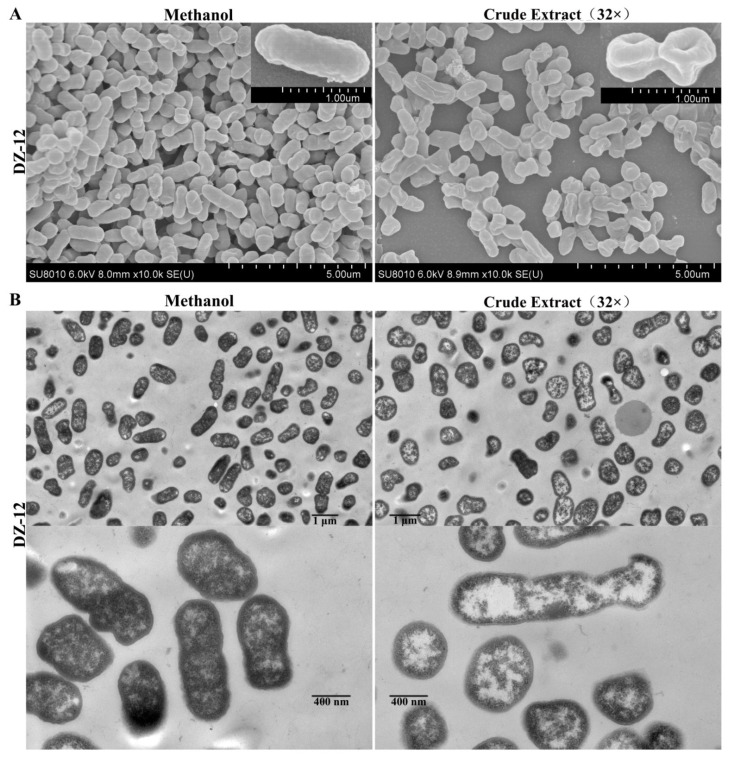
Effect of crude extract on the micro- and ultra-structures of the *P. ananatis* DZ-12 cells, as determined by SEM (**A**) and TEM (**B**).

From the results, pyoluteorin presenting in crude extract contributes to anti-biofilm and ROS generation activities. Thus, the crude extract was directly used in the following experiments. Scanning electron microscopy (SEM) and transmission electron microscopy (TEM) were used to observe the ultrastructural and morphological changes in *P. ananatis* DZ-12 cells under the treatment of crude extract. The SEM results showed that control methanol-treated *P. ananatis* DZ-12 cells had a normal shape, complete and fully cylindrical. In contrast, after being treated with a 32 × dilution of crude extract, cells were depressed and shriveled (Figure 6A). These changes may be due to cytoplasm leakage. TEM was used to confirm these observations. In the methanol control, the structure of the bacterial cell was complete, with a uniform cytoplasmic structure. In contrast, the cell walls and cell membranes of the cells treated with crude extract were severely damaged, causing cytoplasmic extravasation and hollowing (Figure 6B). These results indicate that there were active substances in the crude extract that could damage the cell wall and plasma membrane and cause cytoplasm leakage.

### 2.6. Crude Extract Suppresses the Virulence of Brown Rot of Maize Leaves Caused by P. ananatis DZ-12

**Figure 7:**
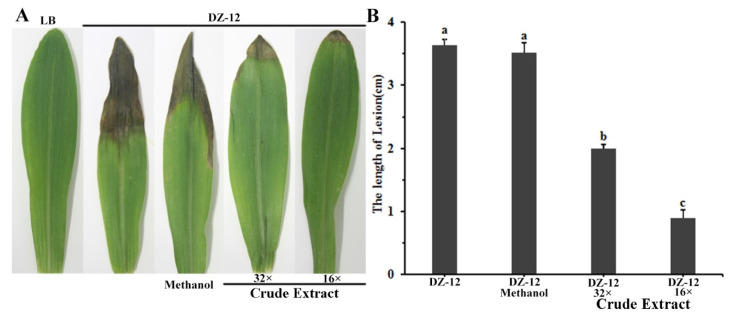
Crude extract from *P. protegens* Pf-5 influenced the infection of *P. ananatis* DZ-12 on maize leaves. (**A**) Disease symptom of maize plants inoculated with the DZ-12 treated with different dilutions of crude extract. Pictures were taken 7 days post-inoculation (dpi) and indicate representative results; (**B**) The graph data show the average lengths of leaf lesions ± standard deviation of three replicates. A concentration of 32× and 16× dilutions of the crude extract from *P. protegens* Pf-5 were used. Different letters represent statistically significant differences according to the one-way ANOVA test (*p* < 0.05).

To study the effect of crude extract on DZ-12 strain pathogenicity on maize seedling leaves, a plant infection experiment was conducted. Different dilutions of crude extract provided significant reductions in the lengths of yellow-brown lesions in maize leaves caused by *P. ananatis* DZ-12. The effect was enhanced with increasing dose concentration (Figure 7). The average lengths of the leaf lesions were 2 cm and 1.0 cm in leaves treated with 32× and 16× dilutions of the crude extract, which were, respectively, 1/2 and 1/4 of the control treatment. The results showed that the crude extract from *P. protegens* Pf-5 reduced the pathogenic effects of *P. ananatis* DZ-12 in maize leaves.

## 3. Discussion

*P. ananatis* causes disease in many important crop plants, including maize, onion, honeydew melon, and pineapple [2,24]. We previously isolated strain DZ-12 from the rotted leaves of maize, and genomic analysis revealed that DZ-12 belongs to *P. ananatis* [2]. In this study, we found that pyoluteorin that is present in the crude extract of *P. protegens* Pf-5 can suppress the growth of *P. ananatis* DZ-12 (Figure 1 and Figure 2). Pyoluteorin is composed of a resorcinol ring, biosynthesized by polyketide synthase/nonribosomal peptide synthase (PKS/NRPS) hybrid complexes involving the sequential addition and modification of simple carboxyl acids to a growing carbon chain [19,25]. A similar study found that pyoluteorin inhibits Oomycetes, including *Pythium ultimum*, and contributes to the biocontrol of *Pythium* damping-off in cotton [26]. Pyoluteorin also has anti-tumor activity, which can induce cell cycle arrest and apoptosis in human triple-negative breast cancer cells [27,28]. Recently, pyoluteorin was reported to have activity against *Chlamydomonas reinhardtii* and *Heterobasidion* species [29,30]. All of these results indicate that pyoluteorin has a broad spectrum of antimicrobial activity. 

The biofilm of *P. ananatis* DZ-12 serves as a major determinant in its pathogenesis [2]. We found that the biofilm of *P. ananatis* DZ-12 was significantly inhibited by a crude extract from *P. protegens* Pf-5 (Figure 3 and Figure 4), and this was confirmed by results from confocal laser scanning microscopy (Figure 4). The further study indicates that pyoluteorin presenting in crude extract contributes to inhibitory effects on the biofilm formation of DZ-12 (Figure 4). The decreased pathogenicity of the cured strain may also be related to its impairment in biofilm formation. The inhibition or prevention of biofilm formation represents an emerging strategy in the war against antibiotic resistance, interfering with key players in bacterial virulence [31]. Pyoluteorin has a pyrrole ring and it belongs to nitrogenous heterocyclic compounds. Several compounds having a similar structure were reported for interesting anti-biofilm properties [32,33].

The crude extract of *P. protegens* Pf-5 induced a high ROS accumulation in DZ-12 cells (Figure 5). Other studies have found that low ROS concentrations act as intracellular messengers for molecular events, but that large amounts of ROS cause cell death [23]. The results also showed that the existence of pyoluteorin in the crude extract resulted in the ROS generation of DZ-12 (Figure 5). Pyoluteorin was reported to induce the generation of intracellular ROS in human cancer cells [28]. Other research showed that pyoluteorin induced cytosolic Ca^2+^ fluxes in *C. reinhardtii* and inhibited its growth [29]. In our previous study, we found that the lipopeptide bacillomycin D produced by *Bacillus amyloliquefaciens* could induce the ROS generation of *F. graminearum* [34]. 

Ultrastructural alterations in the morphologies of DZ-12 were observed using SEM and TEM to investigate the activities of crude extracts. SEM and TEM showed that untreated *P. ananatis* cells were in a normal state, complete with a cylindrical trunk (Figure 6). However, the cells treated with crude extract were depressed and shriveled. This may have been caused by the pyoluteorin present in the crude extract. Recently, a similar phenomenon was also reported. Pyoluteorin could cause a variety of morphological changes in *C. reinhardtii*, such as uniflagellar structures [29]. In human cancer cells, pyoluteorin induces cell cycle arrest, possibly via the reduction of the mitochondrial membrane [28]. 

Lipopeptide compounds are essential in the biocontrol of *Pseudomonas* BCA in plant pathogens. They may be useful for managing plant pathogen infestations because they are selectively nontoxic, stable, and environmentally benign [35]. Our study also showed that orfamide A had bio-surfactant activity (Figure 2). The bio-surfactant activity of cyclic lipopeptides (CLPs) has been reported to contribute to the swarming motility of *Pseudomonas* strains, and of biofilm formation [36] and colonization [37]. Orfamides are CLPs that consist of a saturated or unsaturated unbranched β-hydroxy fatty chain (12, 14, or 16 carbons in length) bound to a decapeptide, with eight of the amino acids forming a lactone ring structure [20]. Orfamide can inhibit the growth of the green algae, *C. reinhardtii* [38]. Orfamide can also act as an insecticidal metabolite against the aphid *Myzus persicae* [39]. However, in this study, orfamide A had no effect on the growth of DZ-12. This is in view of the fact that many pesticides also contain surfactants that increase the solubility of active substances and increase their surface adherence. Thus, the crude extract containing the bio-surfactant orfamide was directly used. 

In conclusion, we demonstrated that the crude extract from *P. protegens* Pf-5 had strong antibacterial activity against the pathogen *P. ananatis* DZ-12 and exhibited significant surface activity. HPLC analysis showed that the polyketide pyoluteorin is present in the crude extract, and it plays an important role in the growth suppression of DZ-12, in vivo and in vitro. This compound has the potential to be developed as a pathogen control agent.

## 4. Materials and Methods

### 4.1. Bacterial Strains and Growth Conditions

The strain *P. protegens* Pf-5 and its mutants were cultured at 30 °C on King’s B (KB) medium B agar. The *gfp*-labeled strain *P. ananatis* DZ-12 was grown at 30 °C on Luria Bertani (LB) medium. Antibiotics ampicillin (100 μg/mL) and chloramphenicol (25 μg/mL) were added into the liquid medium or solid agar plate medium, when required. All of the strains used in this study are listed in Table S1. 

### 4.2. Preparation of Crude Extraction from P. protegens Pf-5

The crude extraction from Pf-5 or its mutants was prepared according to the methods described by Hassan et al. [40] and Oni et al. [41] with some changes. Briefly, a single colony of Pf-5 or its mutants was inoculated into 10 mL of KB medium in a 50 mL conical flask, and cultured at 30 °C, 200 rpm for 24 h. Then, 2 mL of this culture was transferred to 200 mL KB medium in a 500 mL flask, followed by incubation for 48 h at 30 °C. The supernatant was collected by centrifugation at 10,000 rpm for 25 min at 4 °C, and the pH was maintained at 2.0 using HCl. The supernatant was stored at 4 °C overnight. The precipitates were collected by centrifugation at 4 °C for 10 min at 12,000× *g*. The collected precipitate was completely suspended by using an appropriate amount of methanol. Then, the pH of the precipitate was adjusted to 7.0 with NaOH, and allowed to stand for 12 h. Finally, the supernatant was collected by centrifugation at 12,000 rpm for 10 min, and was concentrated to 2 mL using a rotary evaporator. The crude extract was obtained by suspending samples in methanol and performing filtration (0.22 μm, Nylon). 

### 4.3. Construction of the Gene Knockout Mutants of P. protegens Pf-5

The construction process of the Δ*pltB* mutant is used as the example. Briefly, 770 bp upstream and 770 bp downstream fragments were amplified by PCR with two pairs of primers, pltB-F1 (*Xba* I) and pltB-R1, as well as pltB-F2 and pltB-R2 (*Hind* III) (Supporting Information Tables S1 and S2), respectively, to obtain the deleted mutant of the *pltB* gene. The pltB-R1 primer was designed to possess the complementary fragment to the pltB-F2 primer. The upstream and downstream PCR products were infused by using overlap PCR with the primers pltB-F1 and pltB-R2. Subsequently, the 1540 bp fusing fragment was digested with *Xba* I and *Hind* III, and cloned into the same sites of the suicide vector pK18mobsacB. The recombinant plasmid pK18-*pltB* was transferred from *E. coli* Top10 to *P. protegens* Pf-5, with the help of *E. coli* Top10 (pRK-2013), through triparental mating. The single cross-over clones were first selected on KB agar, including Amp and Km. After a second homologous recombination event, the Km-sensitive, Amp-resistant, and sucrose-resistant mutants of *pltB* were then screened. The Δ*pltB* mutant with a 7260 bp deletion in the *pltB* ORF (from 56 bp to 7315 bp) was validated by PCR and sequenced using the primers pltB-outF and pltB-outR. In the same way, we also constructed the Δ*ofa*, Δ*phlA*, ΔhcnABC, Δ*rzxB*, Δ*pfl4656*, and Δ*prnA* mutants (Supporting Information Tables S1 and S2). 

### 4.4. Antibacterial Activity Assay 

The antibacterial activities of Pf-5 and its mutants (Δ*ofa*, Δ*phlA*, Δ*pltB*, Δ*hcnABC*, Δ*rzxB*, Δ*pfl4656*, and Δ*prnA*), the crude extract, and three standards (pyoluteorin, 2,4-DAPG, and orfamide A) were determined by the agar diffusion method described previously [42]. Briefly, the *P. ananatis* DZ-12 strain was grown in LB broth medium overnight at 30 °C, 200 rpm. The overnight culture of *P. ananatis* DZ-12 OD_600_ was maintained at 1.0, and 1 mL of dilute suspensions of DZ-12 was mixed into 100 mL melted LB agar medium, and poured into 9 cm plates. Sterilized filter paper disks 0.6 cm in diameter were placed in 1/4 Petri plates. Then, 5 μL of bacterial suspension (OD_600_ = 2), or corresponding secondary metabolites, was added onto the filter paper. The same amount of methanol was used as a control. The plates were incubated at 30 °C for 12 h, and the plates were then photographed, and the diameter of the inhibition zone was measured. Standard pyoluteorin (Cas No. 25683-07-2) and 2,4-DAPG (Cas No. 2161-86-6) were purchased from Apexbio (Houston, TX, USA). Orfamide A (Cas No. 939960-34-6) was purchased from Bioaustralis (Smithfield, NSW, Australia). 

### 4.5. Bio-Surfactant Activity Assay

Bio-surfactant activity was analyzed by using the expelling oil method previously described by Liang et al. [21]. To obtain the red oil, a 10 mL light fraction of paraffin oil (CAS: 8042-47-5, Sangon Biotech Co., Ltd., Shanghai, China) was dyed red by using 10 mg Sudan III. The 1 mL volume of red oil was dropped onto the 9 cm plate with 20 mL double distilled water (ddH_2_O). After spreading the red oil across the whole surface, 1 μL crude extract was applied to the center of the plate, with 1 μL methanol being used as a control. A total of 1 μL of 875 μg/mL pyoluteorin, 180 μg/mL 2,4-DAPG, and 1030 orfamide A μg/mL were also detected by using the same method. 

### 4.6. Identification of Compounds Using High-Performance Liquid Chromatography Analysis 

The crude extract was added to semi-preparative high-performance liquid chromatography HPLC (Waters, Milford, MA, USA) equipped with a C_18_ column (ZORBAX SB-C18, 4.6 × 250 mm, 5 μm, Agilent, Palo Alto, CA, USA). The mobile phase comprising A was acetonitrile (ACN), with 0.1% (vol/vol) trifluoroacetic acid (TFA); Mobile B was Milli-Q water with 0.1% (vol/vol) TFA. The flow rate was 1 mL/min, and the injection volume for each sample was 5 μL. Pyoluteorin and 2,4-DAPG were detected by a gradient elution procedure: 5–95% ACN for 20 min, held at 95% CAN for 5 min. The UV absorptions at 310 nm and 270 nm were recorded for pyoluteorin and 2,4-DAPG, respectively. For the detection of orfamide A, the sample was eluted from 60–90% ACN in 10 min and held at 90% for 20 min. The detection was processed under UV absorption at 205 nm.

### 4.7. Observed Structural Changes in P. ananatis DZ-12 Caused by Crude Extract

Scanning electron microscopy (SEM) and transmission electron microscopy (TEM) were used to observe the ultrastructural and morphology changes of DZ-12 cells treated with crude extract according to the previous method [31]. For SEM, the cells of DZ-12 were prefixed with 2.5% glutaraldehyde and followed by three rounds of rinsing using 100 mM phosphate buffer. Subsequently, the samples were post-fixed for 3 h in 1% osmium tetroxide, and dehydrated using an ethanol gradient. After that, the samples were coated with gold particles and observed via Hitachi S-3000N SEM at a 5 kV voltage (Hitachi, Tokyo, Japan). For TEM analysis, the prefixed cells were embedded in Epon 812, sectioned using an ultra-microtome, and examined with a Hitachi H-600 transmission electron microscope.

### 4.8. Biofilm Formation

The biofilm formation of *P. ananatis* DZ-12 was initially quantified by staining with crystal violet. The overnight culture of *P. ananatis* DZ-12 OD_600_ reached 0.6, and 200 μL cells were transformed into 20 mL of LB medium. The LB medium contained 16 × or 32× dilutions of crude extract (final concentration), followed by methanol that was used as a control. The bacterial cells were grown at 30 °C, 200 rpm. When the cell OD_600_ reached 1.0, 4 mL of cultured bacterial cells were transferred to a sterilized glass test tube and incubated at 28 °C for 48 h. The bacterial cultures were carefully removed from the glass test tube, and the test tubes were washed gently with ddH_2_O 2–3 times. Finally, the biofilms on the inner surface of glass tubes were stained with 0.1% crystal violet and quantified at 570 nm. The experiment was repeated thrice. 

For the common observation of biofilm formation, a 24-well plate were used. When the OD_600_ of the *P. ananatis* DZ-12 cells reached 0.6, 3 μL of bacterial cultures were, respectively, inoculated into the wells. Each well contained 1.5 mL, with a different dilution of crude extract (16×, 32×) from Pf-5 and its mutants Δ*ofa*, Δ*pltB*, and Δ*phlA.* Three pure commercial compounds were used. The final concentration of pyoluteorin, orfamide A, and 2,4-DAPG, were, respectively, 27 μg/mL, 32 μg/mL, and 12 μg/mL. The 24-well plate was incubated at 28 °C for 48 h. The biofilm was observed. 

In addition, we used the *gfp* gene-labeled strain DZ-12 to understand detailed observations of the biofilm previously described by [43]. A 24-well plate and Nunc Lab-Tek™ II chamber slides were used. Briefly, when the OD_600_ of the *P. ananatis* DZ-12 cells reached 0.6, 3 μL of bacterial culture was inoculated into the wells. Each well contained 1.5 mL, using a different dilution of crude extract (16×, 32×, and 64×). In the control treatment, equal volumes of methanol were added. Then, the wells were gently mixed, and 200 μL of corresponding mixtures were transferred to the Nunc Lab-Tek™ II chamber slides. The 24-well plate and the chamber slides were incubated at 28 °C for 48 h. To photograph the biofilms in the chamber slides, 24-well plates was observed using a Leica SP5 confocal microscope (Leica Microsystems Vertrieb GmbH, Wetzlar, Germany) equipped with Leica LAS AF software. 

### 4.9. Reactive Oxygen Species Detection

The detection of reactive oxygen species (ROS) was performed according to the method previously described [34], with some modifications. Briefly, *P. ananatis* DZ-12 were exposed to a crude extract or 27 μg/mL pyoluteorin, 32 μg/mL orfamide A, and 12 μg/mL 2,4-DAPG, followed by incubation for 4 h at 28 °C. The DZ-12 bacterial cells were collected using centrifugation at room temperature, and then washed two times using 10 mM sodium phosphate buffer (PBS) (pH 7.4). The collected bacterial cells were resuspended using the appropriate amount of PBS to obtain OD_600_ = 1.0 of the bacterial suspension. Then, the bacterial cells were incubated with 10 μM dichloro-dihydro-fluorescein diacetate (DCFH-DA) (JianCheng Bioengineering, Nanjing, China) for 30 min at 19 °C–21 °C. ROS were measured on the basis of the intracellular peroxide-dependent oxidation of DCFH-DA to form the fluorescent compound 2′,7′-dichlorofluorescein (DCF). The samples were observed under fluorescence microscopy (Olympus BX43 microscope excitation 488 nm, emission 535 nm), and analyzed using cell Sens Standard Software v.6.2 (Tokyo, Japan). Fluorescence intensity was determined using an EnSight Multimode Plate Reader (Perkinelmer, Waltham, MA, USA).

### 4.10. Plant Infection Assay

When the OD_600_ of overnight cultured *P. ananatis* DZ-12 cells reached 0.6, the cultured cells (1%) were transferred into 20 mL of LB broth medium with various dilutions (16× or 32×) of crude extract, and grown at 28 °C, 200 rpm for 12 h. The bacterial cells were harvested by centrifugation and washed three times with PBS, and they were resuspended in LB medium to 1 × 10^7^ cells/mL. Then, the bacterial cell suspension was sprayed onto the 7 days old leaves of maize seedlings (c.v B73 cultivar), and PBS was used as a negative control. The plants were maintained in a greenhouse at 30 °C during the evaluation period, with natural light. The development of disease symptoms was monitored and photographs were taken 7 days post-inoculation (dpi). The pathogenicity test assays were repeated in triplicate, with 12 technical replicates. 

### 4.11. Statistical Analysis

A completely randomized design was used to conduct all experiments in the present study, with each experiment repeated thrice. A statistical analysis was performed by using the statistical package SPSS. Tukey’s HSD test was applied for the separation of means at *p* ≤ 0.05, after conducting the analysis of variance (ANOVA) for all data sets.

## Data Availability

Not applicable.

## Supplementary Materials

The following supporting information can be downloaded at: https://www.mdpi.com/article/10.3390/ijms23126431/s1.
