# Supplementary material for: The Role of Pyoluteorin from Pseudomonas protegens Pf-5 in Suppressing the Growth and Pathogenicity of Pantoea ananatis on Maize
Source: Int J Mol Sci. 2022 Jun 9;23(12):6431. doi: 10.3390/ijms23126431 (PMC9223503; doi:10.3390/ijms23126431)
Supplement: Supplementary file 1 [file ijms-23-06431-s001.zip › ijms-1676475-supplementary.pdf]

Table S1 Strains and plasmids used in this study

Strains/Plasmids	Characteristics	Source
P. protegens		
Pf-5	Wild type, Ap^r	[19]
Δ*ofaA*	Pf-5 deleted the *ofaA* gene, Ap^r	This study
Δ*pltB*	Pf-5 deleted the *pltB* gene, Ap^r	This study
Δ*phlA*	Pf-5 deleted the *phlA* gene, Ap^r	This study
Δ*prnA*	Pf-5 deleted the *prnA* gene, Ap^r	This study
Δ*rzxB*	Pf-5 deleted the *rzxB* gene, Ap^r	This study
Δ*hcnABC*	Pf-5 deleted the *hcnABC* gene, Ap^r	This study
Δ*pFL4656*	Pf-5 deleted the *pFL4656* gene, Ap^r	This study
P. ananatis		
DZ-12	Wild-type	[2]
DZ-12(*gfp*)	Wild-type transformed with pBBR-GFP, Gen^R	[2]
E. coli		
Top10	*mcr*A Δ(*mrr-hsdRMS-mcrBC*)ф80*lacZ*ΔM15 Δ*lacX74* *recA1* *araD139* Δ(*ara-leu*)7697 *galU* *galK* *rpsL*(Str^r) *endA1* *nupG*	TaKaRa Company
**Plasmids**		
pK18mobsacB	Broad-host-range gene replacement vector; *sacB*, Km^r	Laboratory stock
pRK-2013	Helper plasmid for triparental mating; Km^r	Laboratory stock
pK18-*ofaA*	pK18mobsacB with *Sal* I-*Hind* III insert of 330 bp and 308 bp segments flanking *ofaA*, Km^r	This study
pK18-*pltB*	pK18mobsacB with *Xba* I-*Hind* III insert of 770 bp and 770 bp segments flanking *pltB*, Km^r	This study
pK18-*phlA*	pK18mobsacB with *Xba* I-*Hind* III insert of 542 bp and 512 bp segments flanking *phlA*, Km^r	This study
pK18-*prna*	pK18mobsacB with *Xba* I-*Hind* III insert of 770 bp and 770 bp segments flanking *prna*, Km^r	This study
pK18-*rzxB*	pK18mobsacB with *Xba* I-*Hind* III insert of 740 bp and 733 bp segments flanking *rzxB*, Km^r	This study
pK18-*hcnABC*	pK18mobsacB with *Xba* I-*Hind* III insert of 452 bp and 518 bp segments flanking *hcnABC*, Km^r	This study
pK18-*pFL4656*	pK18mobsacB with *Xba* I-*Hind* III insert of 566 bp and 529 bp segments flanking *pFL4656*, Km^r	This study

Table S2 DNA primers used in this study

Primer	Primer sequence（5’→3’）
**For mutants construction**	
ofa- F1	ACGCGTCGACTGGGTCAAGCCCTTGCGA (*Sal* I)
ofaA-R1	TGACATGGGAAGCCGGGAGGCGGAAAAATGCGTCAT
ofaA-F2	ATGACGCATTTTTCCGCCTCCCGGCTTCCCATGTCA
ofaA-R2	CCCAAGCTTAGCAGGTCCTCCTGTTCC (*Hind* III)
ofaA-outF	AAATCGCTGATCGATGCGC
ofaA-outR	TCCGGGGTGATCCTGAAGG
pltB-F1	CTAGTCTAGATCGACTACATCTTCATTCACT (*Xba* I)
pltB-R1	ATCAGCTCGGCCCGTGACAATCCACTCCCGATGATTGCAA
pltB-F2	TTGCAATCATCGGGAGTGGATTGTCACGGGCCGAGCTGAT
pltB-R2	CCCAAGCTTGTGCAGGGCCACCAGGGAT (*Hind* III)
pltB-outF	AAGGCCAAGATGGTGGTGG
pltB-outR	CGCTTGAGCGCAAGGATCA
phlA-F1	CTAGTCTAGAGGAAGTGAGAATGGCTTTA (*Xba* I)
phlA-R1	CTTGCGTAGACAGGCGTAAGTTCATTTTCCTCTTGATTCC
phlA-F2	GGAATCAAGAGGAAAATGAACTTACGCCTGTCTACGCAAG
phlA-R2	CCCAAGCTTATAGCCGAACTTCTGGAAG (*Hind* III)
phlA-outF	TAAACCTCGGCGATCAACG
phlA-outR	CGCTGTTCTTCAACACTTCC
prna-F1	CTAGTCTAGACTGGCCCATGACGGCCCG (*Xba* I)
prna-R1	TTCCTGAGCCGCGAGCGTGCGGTGCCGCCGCCCACG
prna-F2	CGTGGGCGGCGGCACCGCACGCTCGCGGCTCAGGAA
prna-R2	CCCAAGCTTTTCTCGATAAGCTGGGTCGTCC (*Hind* III)
prna-outF	GCTTCGTGGCCATGGAAAT
prna-outR	CGCAGCAGGACCTTGAAGA
rzxB- F1	CTAGTCTAGACGTTGTCTTGCAGCAAGCG (*Xba* I)
rzxB-R1	GCGCTGGTGAAGGACATCAGAGCTCGCGCAGGTAGTGG
rzxB-F2	CCACTACCTGCGCGAGCTCTGATGTCCTTCACCAGCGC
rzxB-R2	CCCAAGCTTCGGACAAACCTTCATCCCACT (*Hind* III)
rzxB-outF	TTGCCATTGGCAATTTTTCTG
rzxB-outR	CTCGATCAGCACCTGATTG
hcnABC-F1	CTAGTCTAGAGCCGTGGTCTGGCTTGAAA (*Xba* I)

hcnABC-R1	GCCAGCGCCTACAGAGCGGTGTGGGTTCATCCGTGAAAA
hcnABC-F2	TTTTCACGGATGAACCCACACCGCTCTGTAGGCGCTGGC
hcnABC-R2	CCCAAGCTTCATCGACTACCTGGAAACCC (*Hind* III)
hcnABC-outF	TGGAAGAAGCCAAGCAGGC
hcnABC-outR	CTGCCCTTCGAGCATCACT
pFL4656-F1	CTAGTCTAGACCATTTTTCAGCAACCCGCA (*Xba* I)
pFL4656-R1	GGCGTTCATGGAGGTTTCTGATATCCCTGCCTTGTTGTT
pFL4656-F2	AACAACAAGGCAGGGATATCAGAAACCTCCATGAACGCC
pFL4656-R2	CCCAAGCTTAGGTCTTCTGGATGTGCTCC (*Hind* III)
pFL4656-outF	CTGATCATCGAGCCGATCCA
pFL4656-outR	TACAGCAGGATGCCGAGAT

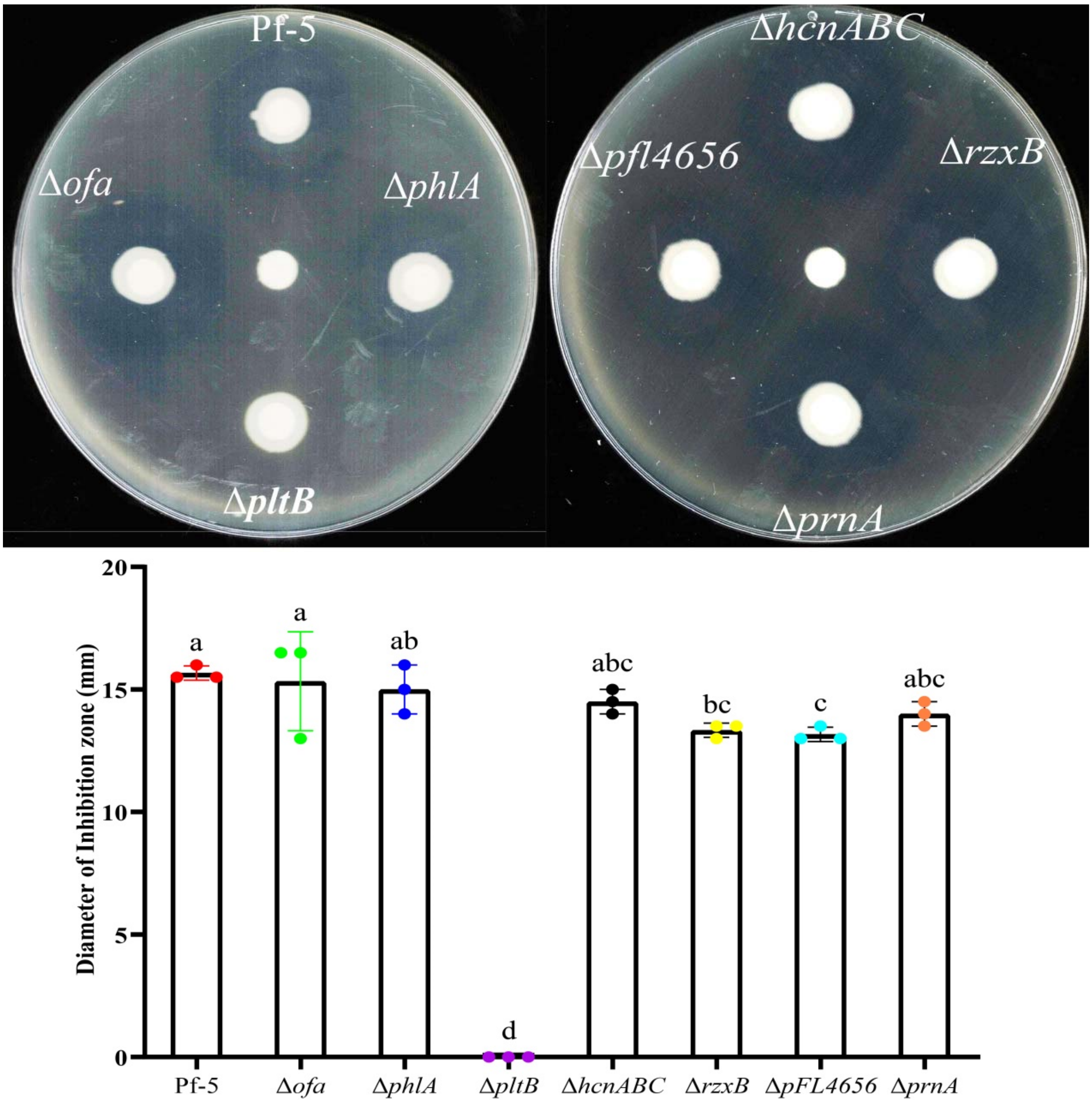

Figure S1 The antibacterial activity detection of Pf-5 and its mutants. The graph data show the average diameter (mm) ± standard deviation (SD) of three replicates. Different letters represent statistically significant differences according to the one-way ANOVA test ($P < 0.05$).
